# Supplementary material for: The Sclerotinia sclerotiorum ADP-Ribosylation Factor 6 Plays an Essential Role in Abiotic Stress Response and Fungal Virulence to Host Plants
Source: J Fungi (Basel). 2023 Dec 25;10(1):12. doi: 10.3390/jof10010012 (PMC10817261; doi:10.3390/jof10010012)
Supplement: Supplementary file 1 [file jof-10-00012-s001.zip › jof-2750422-supplementary.pdf]

Journal of *Fungi*

Supplementary Information: The *Sclerotinia sclerotiorum* ADP-Ribosylation Factor 6 Plays an Essential Role in Abiotic Stress Response and Fungal Virulence to Host Plants

Kunmei Wang, Siyi Wang, Ting Wang, Qi Xia and Shitou Xia *

Hunan Provincial Key Laboratory of Phytohormones and Growth Development, College of Bioscience and Biotechnology, Hunan Agricultural University, Changsha 410128, China; yuhunan@stu.hunau.edu.cn (K.W.); wangsiyi@stu.hunau.edu.cn (S.W.); tina@stu.hunau.edu.cn (T.W.)

* Correspondence: xstone0505@hunau.edu.cn

Citation: Wang, K.; Wang, S.; Wang, T.; Xia, Q.; Xia, S. The *Sclerotinia sclerotiorum* ADP-Ribosylation Factor 6 Plays an Essential Role in Abiotic Stress Response and Fungal Virulence to Host Plants. *J. Fungi* **2024**, *10*, 12. https://doi.org/10.3390/jof10010012

Academic Editors: Zonghua Wang and Jun Huang

Received: 16 November 2023
Revised: 21 December 2023
Accepted: 22 December 2023
Published: 25 December 2023

Copyright: © 2023 by the authors. Licensee MDPI, Basel, Switzerland. This article is an open access article distributed under the terms and conditions of the Creative Commons Attribution (CC BY) license (https://creativecommons.org/licenses/by/4.0/).

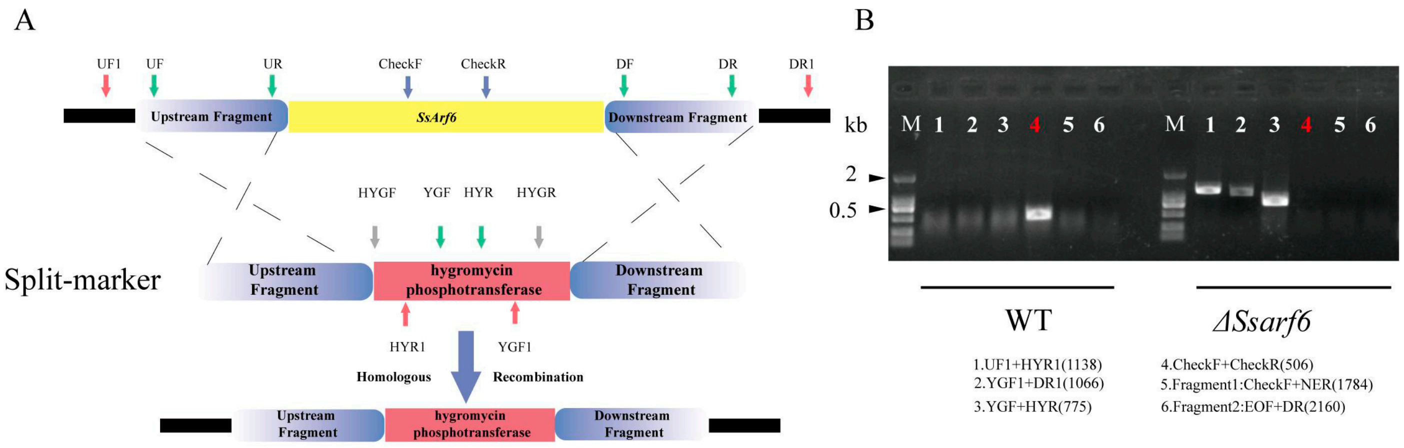

Figure S1. Knockout of *SsArf6* in *S. sclerotiorum.* **(A)** The schematic diagram of the strategy used for knockout of *SsArf6*. The *SsArf6* gene, Hygromycin phosphotransferase gene (hph), and G418 resistance gene *NEO* were denoted by yellow, red, and green rectangles, respectively. The labeled primers in the diagram were used for amplifying flanking sequences of *SsArf6* or facilitating mutant screening. **(B)** The verification of knockout of *SsArf6* by PCR. Genomic DNA obtained from WT, knockout mutant *ΔSsarf6* were utilized as templates for PCR. A total of six primer pairs were employed to detect the insertion of *hph* and its upstream and down-stream fragments, as well as to confirm the knockout of *SsArf6*. The sizes of the amplified bands were indicated within brackets. The lanes labeled as M represent the DNA marker.

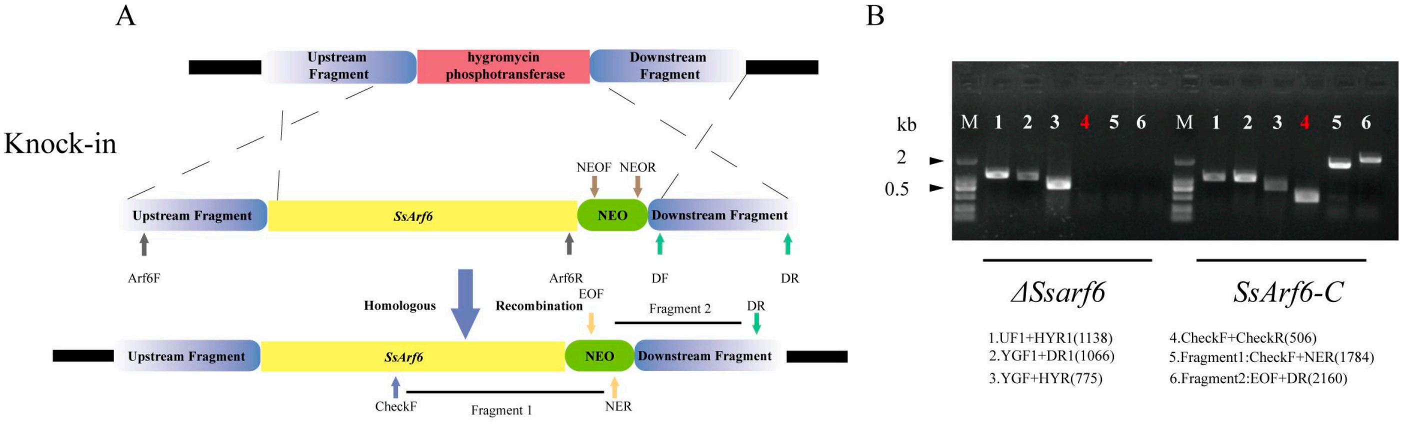

Figure S2. Complementation of *SsArf6* in *ΔSsarf6* mutants of *S. sclerotiorum.* **(A)** The schematic diagram of the strategy used for the complementation of *ΔSsarf6*. The *SsArf6* gene, *Hygromycin phosphotransferase* gene (hph), and G418 resistance gene *NEO* were denoted by yellow, red, and green rectangles, respectively. The labeled primers in the diagram were used for amplifying full-length genomic DNA of *SsArf6* or facilitating transformant screening. **(B)** The verification of complementation of *SsArf6* by PCR. Genomic DNA obtained from the deletion mutant *ΔSsarf6*, and complemented mutant *SsArf6-C* were utilized as templates for PCR. A total of six primer pairs were employed to confirm the complementation of *SsArf6*. The sizes of the amplified bands were indicated within brackets. The lanes labeled as M represent the DNA marker.

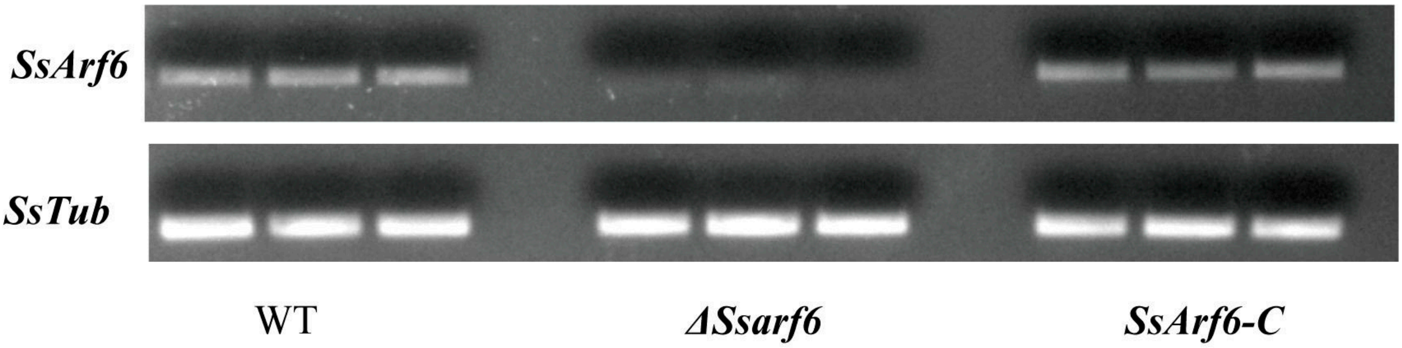

Figure S3. The semi-quantitative RT-PCR analysis of the WT, *ΔSsarf6* and *SsArf6-C* strains. Semi-quantitative RT-PCR was performed using cDNA templates from WT, *ΔSsarf6* and *SsArf6-C* for 28 cycles. *SsTub* (β-tubulin) was used as an internal reference.

Table S1. Primers used in study

Primer name	Primer sequence	Primer function
UF	GACAATCCGAGCACTCAA	Amplification of upstream sequence of *SsArf6*. The red part presents homologous sequences from HY fragment using for homologous recombination.
UR	GTGCTCCTTCAATATCATCTTCTGT CGAGATATGCGTGTGTATG	
DF	AGGTACACTTGTTTAGAGGTAATCCGCTCCAATCTACCTTCTCG	Amplification of upstream sequence of *SsArf6*. The red part presents homologous sequences from YG fragment using for homologous recombination.
DR	GGAATTGGAAAATGGGGT	
HYGF	ACAGAAGATGATATTGAAGGAGCAC	Amplification of HY and YG fragment
HYR	GCATCATCGAAATTGCCGTCAACC	
YGF	TCTCGGAGGGCGAAGAATCTCGTGC	
HYGR	GGATTACCTCTAAACAAGTGTACCT	
UF1	GACTCTTACCTCGCAATGAA	Check if the position of homologous substitution is correct
HYR1	CGTTCCTGTCTGCTAATAAG	
YGF1	TAGTGAATGCTCCGTAACA	
DR1	CTCGGTCATTGATTGTGTATAG	
Arf6F	GACTCTTACCTCGCAATGAA	Amplification of upstream and full-length sequences of *SsArf6*.The lowercase part presents homologous sequences from NEO fragment using for homologous recombination.
Arf6R	agtgctccttcaatatcatcttctgCTTTGCTTGTGGAGCAGG	
NEOF	CAGAAGATGATATTGAAGGAGCAC	Amplification of NEO sequence of G418 resistance gene
NEOR	GGATTACCTCTAAACAAGTGTACCT	
checkF	GTAGAGACGGTGACATATAAGA	Knockout transformants identification
checkR	CATACCAATCCTTCCATTAACC	
checkF	GTAGAGACGGTGACATATAAGA	Complementation transformants identification
NER	CGTCAAGAAGGCGATAGAA	
EOF	TCTCCTGTCATCTCACCTT	
DR	GGAATTGGAAAATGGGGT	
SsTubqF	ACCTCCATCCAAGAACTC	Amplification of reference gene
SsTubqR	GAACTCCATCTCGTCCAT	
